# Characterizing the Peroxisome Proliferator-Activated Receptor (PPAR**γ**) Ligand Binding Potential of Several Major Flame Retardants, Their Metabolites, and Chemical Mixtures in House Dust

**DOI:** 10.1289/ehp.1408522

**Published:** 2014-10-14

**Authors:** Mingliang Fang, Thomas F. Webster, P. Lee Ferguson, Heather M. Stapleton

**Affiliations:** 1 Nicholas School of the Environment, Duke University, Durham, North Carolina, USA; 2 Department of Environmental Health, Boston University School of Public Health, Boston, Massachusetts, USA

## Abstract

Background: Accumulating evidence has shown that some environmental contaminants can alter adipogenesis and act as obesogens. Many of these contaminants act via the activation of the peroxisome proliferator-activated receptor γ (PPARγ) nuclear receptor.

Objectives: Our goal was to determine the PPARγ ligand binding potency of several major flame retardants, including polybrominated diphenyl ethers (PBDEs), halogenated phenols and bisphenols, and their metabolites. Ligand binding activity of indoor dust and its bioactivated extracts were also investigated.

Methods: We used a commercially available fluorescence polarization ligand binding assay to investigate the binding potency of flame retardants and dust extracts to human PPARγ ligand-binding domain. Rosiglitazone was used as a positive control.

Results: Most of the tested compounds exhibited dose-dependent binding to PPARγ. Mono(2-ethylhexyl) tetrabromophthalate, halogenated bisphenols and phenols, and hydroxylated PBDEs were found to be potent PPARγ ligands. The most potent compound was 3-OH-BDE-47, with an IC_50_ (concentration required to reduce effect by 50%) of 0.24 μM. The extent of halogenation and the position of the hydroxyl group strongly affected binding. In the dust samples, 21 of the 24 samples tested showed significant binding potency at a concentration of 3 mg dust equivalent (DEQ)/mL. A 3–16% increase in PPARγ binding potency was observed following bioactivation of the dust using rat hepatic S9 fractions.

Conclusion: Our results suggest that several flame retardants are potential PPARγ ligands and that metabolism may lead to increased binding affinity. The PPARγ binding activity of house dust extracts at levels comparable to human exposure warrants further studies into agonistic or antagonistic activities and their potential health effects.

Citation: Fang M, Webster TF, Ferguson PL, Stapleton HM. 2015. Characterizing the peroxisome proliferator-activated receptor (PPARγ) ligand binding potential of several major flame retardants, their metabolites, and chemical mixtures in house dust. Environ Health Perspect 123:166–172; http://dx.doi.org/10.1289/ehp.1408522

## Introduction

According to a report from the Centers for Disease Control and Prevention (Ogden et al. 2012), 17% of children between 2 and 19 years of age in the United States are obese, and health care costs associated with obesity in the United States in 2008 were estimated to be > $140 billion. Although genetics, diet, and exercise all contribute to obesity, recent studies have shown that prenatal exposures to “environmental obesogens,” including bisphenol A, phthalates, organotins, and perflourinated compounds may increase the risk of obesity in children (Janesick and Blumberg 2011). Several studies have found significant associations between urinary metabolites of phthalates and obesity (Wang et al. 2013). High levels of several persistent organic pollutants [e.g., DDE (dichlorodiphenyldichloroethylene), hexachlorobenzene, and polybrominated diphenyl ethers (PBDEs)] have also been found to be associated with obesity in humans (Tang-Péronard et al. 2011).

Current research suggests that several of the obesogenic compounds act via a mechanism involving activation of peroxisome proliferator-activated nuclear receptors (PPARs) during perinatal development (Janesick and Blumberg 2011). PPARs are master transcriptional regulators controlling intracellular lipid flux and adipocyte proliferation and differentiation. Heterodimerized with the retinoid X receptor, PPARs serve as metabolic ligand sensors for a variety of hormones, dietary fatty acids, and their metabolites (Grün and Blumberg 2009). Chemicals that specifically activate PPARγ and up-regulate expression may promote the development of obesity. Studies investigating the crystal structure of PPARγ with thiazolidinedione drugs have found that it exhibits flexible plasticity in the ligand-binding domain (LBD), which allows it to accommodate a wide variety of ligands (Nolte et al. 1998). The endogenous ligands of PPARγ include polyunsaturated fatty acids, prostanoids, and oxidized fatty acids. Several antidiabetic drugs of the thiazolidinedione class, such as rosiglitazone, target PPARγ (Lu and Cheng 2010), and weight gain is often a side effect (Ness-Abramof and Apovian 2005). Environmental contaminants including tributyltin (TBT), triphenyltin (TPT), and mono(2-ethylhexyl) phthalate (MEHP) [a metabolite of the phthalate di(2-ethylhexyl)phthalate (DEHP)], have been shown to up-regulate and stimulate several PPARs (Feige et al. 2007).

Flame retardants (FRs) are a class of compounds that have been used in large volumes over the past few decades to reduce the flammability of textiles, polymers, and resins. Accumulating evidence has suggested that FRs might represent an important class of compounds that could bind to PPARγ and disrupt signaling. A recent study found that 2,2´,6,6´-tetrabromobisphenol A (TBBPA) and 3,3´,5,5´-tetrachlorobisphenol A (TCBPA), were agonists of PPARγ (Riu et al. 2011). In our recent studies, Firemaster® 550 (FM550), a FR replacement for pentabromodiphenyl ethers (pentaBDEs), activated PPARγ and initiated adipocyte differentiation *in vitro* (Pillai et al. 2014), which may explain why perinatal exposure to FM550 in rats led to obesity and glucose sensitivity (Patisaul et al. 2013). Therefore, further investigation of PPARγ-targeted disruption by FRs is warranted.

Several organophosophate compounds are also structurally similar to PPARγ exogenous agonists. For example, tributylphosphate (TBuP) and tris(2-butoxyethyl) phosphate (TBEP) are structurally similar to TBT. The PPARγ ligand triphenyl phosphate (TPP) and its antioxidant analog triphenylphosphite (TPPi) resemble TPT. Many of the PBDE metabolites (i.e., hydroxylated PBDEs and halogenated phenols) are structurally similar to TBBPA, which was shown to be a PPARγ ligand (Riu et al. 2011). Therefore, it would be of great interest to investigate whether these structurally similar compounds could act on PPARγ.

Indoor dust is a primary sink for additive chemicals applied to consumer products, and many of the reported environmental obesogens are found abundantly in house dust. For example, Rudel et al. (2003) detected DEHP in all dust samples analyzed, with a geometric mean concentration of 340,000 ng/g; organotins are also commonly detected (Kannan et al. 2010). Three of the four chemicals in FM550 were widely detected in house dust samples in the United States (Dodson et al. 2012; Stapleton et al. 2014). Young children in the United States spend most of their time (> 95%) indoors where they are chronically exposed to FRs due to increased hand to mouth activity [U.S. Environmental Protection Agency (EPA) 2009]. Therefore, it is important to investigate the PPARγ binding potency of environmentally relevant house dust samples.

Little attention has been given to the effect of bioactivation on PPARγ disruption. Several studies have revealed that metabolites can be more potent endocrine disruptors than the parent compounds. For example, the metabolite MEHP exhibited much stronger PPARγ binding potency than its parent compound, DEHP (Feige et al. 2007). Springer et al. (2012) reported that tetrabromo mono(2-ethylhexyl)phthalate (TBMEHP), a metabolite of bis(2-ethylhexyl) tetrabromophthalate (TBPH), was an agonist for PPARs in mouse NIH 3T3 L1 preadipocyte cells, whereas TBPH was not. The chemicals present in ingested house dust are absorbed into the digestive system and can be metabolized to chemicals with more polar functional groups. Therefore, it is important to determine whether the PPARγ binding potency of contaminants changes with metabolism.

The primary goals of the present study were to *a*) characterize the binding potency of several major FRs, such as PBDEs (and their metabolites), using a human protein–ligand binding assay; *b*) test the PPARγ binding activity of indoor dust extracts; and *c*) examine the effect of *in vitro* bioactivation on the PPARγ binding potency of dust extracts.

## Materials and Methods

*Chemicals*. The tested compounds included FM550 (and their metabolites), several PBDE congeners (and their metabolites), and halogenated phenols and bisphenols. All of the tested compounds and their abbreviations are listed in Supplemental Material, “Abbreviations.” Rosiglitazone and MEHP were used as positive controls. For chemical structures of the tested compounds, see Supplemental Material, Figure S1. We purchased 2,2´,4,4´-tetrabromodiphenyl ether (BDE-47) and 2,2´,4,4´,5-pentabromodiphenyl ether (BDE-99), their metabolites [i.e., 3-OH-BDE-47, 5-OH-BDE-47, 6-OH-BDE-47, 5´-OH-BDE-99, and 6´-OH-BDE-99], and TBBPA (98% purity) from AccuStandard (New Haven, CT). 2,4,6-Tribromophenol (2,4,6-TBP, 99%), 2,4,6-triiodophenol (2,4,6-TIP, 97%), 2,4,6-trifluorophenol (2,4,6-TFP, 99%), 2,4,6,-trichlorophenol (2,4,6-TCP, 98%), TPP (99%), diphenyl phosphate (DPP, 99%), rosiglitazone (98%), triclosan (> 97%), TBT (96%), TBEP (94%), TPPi (97%), dl-dithiothreitol (DTT, > 99%), β-nicotinamide adenine dinucleotide 2´-phosphate reduced tetrasodium salt hydrate (β-NADPH, > 93%), magnesium chloride (hexa-hydrates, > 99%), and dextran (*Leuconostoc* spp.; molecular weight, 6,000–10,000) were purchased from Sigma-Aldrich (St. Louis, MO). We purchased TPT (95%) from Acros Organics (Fairlawn, NJ) and TCBPA (98%) from TCI America (Portland, OR). Tetrabromobenzoic acid (TBBA; estimated > 98% purity by H1-NMR) was synthesized by the Duke Small Molecule Synthesis Facility. TBMEHP was a gift from K. Boekelhide (Brown University, Providence, RI). MEHP (98%) was purchased from Wako Pure Chemical Industrials, Ltd. (Osaka, Japan). A commercial standard of FM 550 was supplied by Great Lakes Chemical (West Lafayette, IN), a company owned by Chemtura (Philadelphia, PA). The ITP commercial mixture was purchased from Jinan Great Chemical Industry Co., Ltd (Commercial Grade; Jinan, PRC). All solvents and other materials were of HPLC grade.

*Chemical analysis*. To investigate the elution profile of chemicals in the gel permeation chromatography (see Supplemental Material, “Operation of Gel Permeation Chromatography”), DEHP, MEHP, TBBPA, TBBA, and other tested compounds were quantitatively analyzed by either liquid chromatography (LC) tandem mass spectrometry (LCMS) (Agilent 6410 Triple Quad LCMS; Agilent Technologies, Santa Clara, CA), or gas chromatography coupled with mass spectrometry detector (GC-MSD). See Supplemental Material, Table S1, for details of the parameters used.

*PPAR*γ *competitive binding assay*. A detailed description of the PPARγ binding assay is provided in Supplemental Material, “PPARγ Competitive Binding Assay and Quality Assurance/Quality Control.” Briefly, we used a commercially available high-throughput ligand binding assay (PolarScreen™ PPARγ-Competitor Assay Kit; Invitrogen, Carlsbad, CA) to investigate the binding potency of the tested compounds to PPARγ LBD. The kit uses the human-derived recombinant PPARγ LBD tagged with an *N*-terminal GST-tag and a selective fluorescent PPARγ ligand (PPARγ Green). Fluorescence polarization (FP) was measured using a SpectraMax M5 plate reader in FP mode with 485-nM excitation and 535-nm emission wavelengths. To measure ligand binding, we quantified polarization value (mP) of the bound protein using the following equation:

*mP* = 10^3^ × (*I*_parallel_ – *I*_perpendicular_) ÷ (*I*_parallel_ + *I*_perpendicular_), [1]

where *I*_parallel_ and *I*_perpendicular_ are the fluorescence intensity of emissions that are parallel and perpendicular, respectively, to the excitation light (Rossi and Taylor 2011).

*Dust sample dosing*. We tested extracts of indoor dust samples (*n* = 23) collected from our previous studies and a dust Standard Reference Material [SRM 2585, Organic Contaminants in House Dust; National Institute of Standards and Technology (NIST), Gaithersburg, MD] for ligand binding potential. The indoor dust samples were investigator collected from the main living areas of homes for groups A (Stapleton et al. 2012) and D (Stapleton et al. 2014). Dust samples in group B were collected from gymnastics studios (Carignan et al. 2013b). Dust samples in group C were investigator collected from office environments (Watkins et al. 2013), and those in group E were participant-collected dust samples from the main living area, as reported by Hoffman et al. (2014). All dust samples were extracted with acetone:hexane (1:1, vol/vol) using sonication, and then concentrated, filtered, and reconstituted in dimethyl sulfoxide (DMSO). Fluorescence background (FB) from the dust matrix was initially observed in the dust extracts (observed by spiking the incubation buffer solution with the extract but without PPARγ LBD and PPARγ Green). Therefore, the dust extracts were cleaned and diluted prior to measuring the PPARγ ligand binding activity. As shown in Supplemental Material, Figure S2A, a FB dose response of SRM 2585 was observed, and dilution greatly reduced the FB from the dust matrix. To clean the extracts, we used gel permeation chromatography (GPC; Environgel GPC system; Waters, Milford, MA), which can partially remove large molecular weight (MW) compounds containing fluorophores (see Supplemental Material, “Operation of Gel Permeation Chromatography” and Table S2). To minimize FB, further dilution was performed until no obvious FB (i.e., < 5% intensity of the complex consisting of 1.25 nM PPARγ Green and 38 nM PPARγ LBD) was observed. Following GPC cleanup and dilution, a single concentration of 3 mg dust equivalent quantity (DEQ) per milliliter PPARγ assay medium was prepared to qualitatively investigate the relative PPARγ binding potency of the dust samples; we examined the full dose response of one potent dust extract. To quantitatively estimate the effect of FB on the polarization values, we spiked the positive control (rosiglitazone, 12.5 μM) into several different dose levels of SRM 2585 extract previously cleaned by GPC to measure the ligand binding activity relative to the pure standard.

*Bioactivation of dust samples*. We assessed the influence of biotransformation on ligand binding activity by incubating dust extracts in pooled liver S9 fractions prepared from Sprague-Dawley rats [Rat (Sprague-Dawley) S9 Fractions; Gibco, Grand Island, NY). Bioactivation was assessed in 7 of the 23 dust samples (1 dust sample was tested in triplicate, while the others were tested once because of dust mass limitations) and in SRM 2585 (*n* = 3). The 7 dust samples were from groups A (samples 5, 7, and 8), B (samples 9 and 10), and C (samples 11 and 12). The influence of biotransformation was also investigated using pure chemical standards. DEHP (100 μM) and a mixture (MIX) containing 1 μM each of FM550, isopropylated triaryl phosphate (ITP), BDE-47, BDE-99, and DEHP were evaluated for binding activity before and after bioactivation. For a detailed description of the method, see Supplemental Material, “Bioactivation of Dust Samples” and Figure S3. Briefly, dust samples were bioactivated by incubation with an S9 fraction (1 mg protein/mL), extracted, and cleaned by dextran-assisted liquid–liquid extraction and phenolic extraction. An additional sample of each dust extract was incubated with inactive S9 fraction (by adding 150 μL of ice-cold 6 M HCl before incubation) to serve as a control. To test the efficacy of metabolism, MEHP, which is a metabolite of DEHP in house dust, was used as a marker compound to optimize the incubation method (see Supplemental Material, Figure S4). To compare the bioactivation difference between rodents and humans, we used a pooled human liver S9 (CellzDirect, Durham, NC) to bioactivate SRM 2585. MEHP, which is a metabolite of DEHP in house dust, was used as a marker compound to optimize the incubation method (see Supplemental Material, “Performance of the Bioactivation of Dust”).

*Data analysis*. IC_50_ (concentration required to reduce effect by 50%) values and dissociation constants were calculated to compare the binding potency. In the competitor study, the dose–response curve was depicted as a ligand-binding, three-parameter sigmoidal dose–response model in the Regression Wizard in SigmaPlot 12.0 (Systat Software Inc., Chicago, IL):

*y* = *min* + (*max – min*)/(1 + 10^(logIC^_^50^_^–^ *^x^*^)^), [2]

where *y* is the measured polarization value (mP); *x* is the log of the compound concentration; *max* is the mP of the DMSO control or the maximum mP of the tested compound; *min* is the the basal mP when reference agonists completely inhibit the binding between PPARγ LBD and PPARγ Green. Because *min* was not zero and varied between batches, high doses of rosiglitazone (10 μM) were run alongside each batch to roughly calculate the *min*_nominal_. The dissociation constants were calculated according to the following equation (Lin et al. 1999):

IC_50_/[PPARγ Green] = *K*_d,ligand_/*K*_d,probe_, [3]

where *K*_d,probe_ is the dissociation constant calculated from titration of 1.25 nM PPARγ Green with added PPARγ LBD concentration.

*Statistical analyses*. All statistical analyses were conducted using SigmaPlot 12.0; all tests were two-tailed, with α = 0.05 considered significant. For comparison of the binding potencies of the dust extracts, all FP values of the dust samples were normalized to the procedural blank. Then a one-way analysis of variance was conducted, and a Newman–Keuls post hoc test was used to identify which dust extracts were significantly different from the procedural control. For comparison of the PPARγ binding activity before and after metabolism, all the data were normalized to the mP of the S9 control, and Student’s *t*-test was used to test the difference between active S9 and inactive S9 for the dust samples with triplicate incubations. For the bioactivated dust (*n* = 6) with single measurements, we used the paired *t*-test. For a description of quality control, see Supplemental Material, “PPARγ Competitive Binding Assay and Quality Assurance/Quality Control.”

## Results

*Performance of the FP assay*. We used rosiglitazone as a positive control in the ligand binding assay. As shown in Table 1, the IC_50_ of rosiglitazone was 0.23 μM; the FP range was > 120 mP, indicating a good dynamic range for the dose response. A PPARγ LBD titration curve was also investigated by varying the protein concentration in 1.25 nM PPARγ Green (see Supplemental Material, Figure S5). In this experiment we used 38 nM of the PPARγ LBD, which was in the linear range of the titration curve, providing a calculated *K*_d_ of 20 nM. A U-shaped dose–response curve was observed for some tested compounds, which probably was a result of limited solubility and precipitation of the compounds. Under such circumstances, the FP values of the concentration on the right side of the U shape were omitted from the data analysis, and partial dose–response curves were analyzed. The primary challenge of this assay was the fluorescence interference from the dust matrix in the extracts. As shown in Supplemental Material, Figure S2A, GPC cleanup can reduce the FB significantly, which suggests that macromolecules might be causing the observed interference. After further dilution, a dose of 3 mg DEQ/mL was used for the dust samples. In the matrix-spiked rosiglitazone test, the binding activity of rosiglitazone was completely masked at a high matrix background (12.5 mg DEQ/mL) (see Supplemental Material, Figure S6). The FB of house dust increased the fluorescence intensity of emission parallel to the excitation plane more than that perpendicular to the excitation plane, which resulted in the increased mP. It is impossible to completely eliminate background interference, and exhaustive cleanup increases the possibility of analyte loss. We estimate that at the dosing concentration used in this study (3 mg DEQ/mL), the binding potency of house dust might actually be underestimated by 5–10% due to the fluorescence interference from the dust matrix. This estimate is based on the difference between the fluorescent signals in dust extracts spiked with and without rosiglitazone (see Supplemental Material, Figure S6). Overall, we conclude that the FP assay was appropriate and efficient to evaluate the binding potency of the tested compounds and dust extracts. The dose–response curves of the tested compounds are shown in Supplemental Material, Figure S7, and the calculated IC_50_ together with *K*_d_ is listed in Table 1.

**Table 1:** IC_50_ values, dissociation constants (*K*_d_), and the relative potency of the studied compounds.

Parent compound/metabolite	IC_50_ (μM)	*K*_d_ (μM)	Relative potency
Rosiglitazone^*a*^	0.23	0.12	1.0000
TBB^*a*^	NA	NA	NA
TBBA	42.0	22.10	0.0055
TBPH^*a*^	NA	NA	NA
TBMEHP	0.64	0.34	0.3594
DEHP^*a*^	NA	NA	NA
MEHP	3.80	2.00	0.0605
TPP^*a*^	40.0	20.87	0.0058
DPP	627.0	327.13	0.0004
ITP^*a*^	60.0	31.30	0.0038
TPT^*a*^	1.72	0.90	0.1337
TPPi^*a*^	> 1,250	> 652.17	< 0.002
TBT^*a*^	0.30	0.16	0.7667
TBuP^*a*^	137.0	71.48	0.0017
TBEP^*a*^	103.0	53.74	0.0022
BPA^*a*^	NA	NA	NA
TCBPA^*a*^	5.18	2.70	0.0444
TBBPA^*a*^	1.49	0.78	0.1544
2,4,6-TFP^*a*^	NA	NA	NA
2,4,6-TCP^*a*^	100.0	52.17	0.0023
2,4,6-TBP^*a*^	36.3	18.94	0.0063
2,4,6-TIP^*a*^	1.84	0.96	0.1250
BDE-47^*a*^	> 12.0	> 6.25	< 0.16
3-OH-BDE-47	0.24	0.13	0.9583
5-OH-BDE-47	3.09	1.61	0.0744
6-OH-BDE-47	> 10.0	> 5.22	< 0.023
BDE–99^*a*^	NA	NA	NA
5´-OH-BDE-99	30.0	15.65	0.0077
6´-OH-BDE-99	> 50.0	> 26.09	< 0.0046
Triclosan	12.5	6.52	0.0184
NA, no effect at 250 μM. The relative potency of rosiglitazone, the positive control, was set at 1.^***a***^Parent compound.			

**Figure 1:**
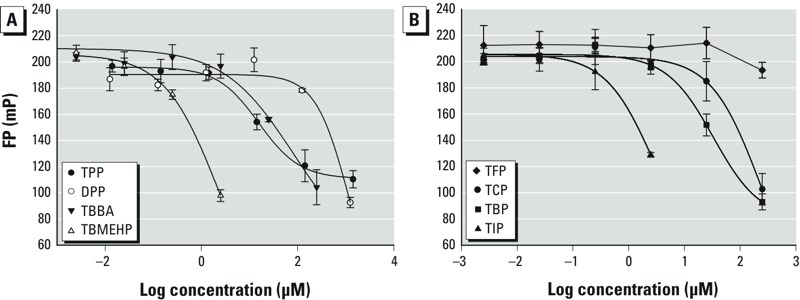
Fluorescence polarization value (mP) of 1.25 nM PPARγ Green as a function of (*A*) TPP, and several FM550 metabolites (DPP, TBBA, and TBMEHP), and (*B*) 2,4,6–TFP, TCP, TBP, and TIP concentration in 40 μL of 38 nM PPARγ LBD. Values shown are the mean ± SD of the triplicates.

*FM550 metabolites*. We recently reported that although the organophosphate components in FM550 did bind to PPARγ, the brominated components, TBB and TBPH, did not (Pillai et al. 2014). Here, we also investigated the binding affinities of potential metabolites of the individual FM550 components (see Figure 1A). The metabolites of TBB and TBPH [TBBA and TBMEHP (Roberts et al. 2012), respectively] bound PPARγ effectively. As shown in Table 1, TBBA was a moderately potent ligand of PPARγ with an IC_50_ of 42 μM. The binding of TBMEHP was particularly potent, with an IC_50_ of 0.64 μM, which was much lower than the well-known PPARγ agonist MEHP (3.8 μM) and comparable to the PPARγ-binding pharmaceutical compound rosiglitazone (IC_50_ = 0.23 μM). The metabolite of TPP (IC_50_ = 40 μM), DPP (IC_50_ = 627 μM), was one order of magnitude less potent than its parent compound.

*Halogenated phenols and bisphenols*. Phenols and biphenol compounds with different degrees of halogenation were also tested for binding with PPARγ. We observed a dose–response relationship for all the tested phenols except 2,4,6-trifluorophenol (2,4,6-TFP). Potency increased with the size of the halogen in the order of fluorine < chlorine (IC_50_ = 100 μM) < bromine (IC_50_ = 36.3 μM) < iodine (IC_50_ = 1.84 μM) (Figure 1B, Table 1). A significant FB was observed for TIP at concentrations > 10 μM. A similar trend in binding with halogenation was observed for TBBPA (IC_50_ = 1.49 μM) and TCBPA (IC_50_ = 5.18 μM), which are known PPARγ ligands; however, BPA did not exhibit any binding. Triclosan, which is largely applied in personal care products, also exhibited PPARγ binding with an IC_50_ of 12.5 μM.

*BDE and BDE metabolites*. The binding activity of BDEs was very poor. The calculated IC_50_ for BDE-47 was > 12 μM, and no binding was observed for BDE-99 at any dose tested. However, some of the OH-BDEs were found to be very potent ligands of PPARγ (Table 1). The BDE-47 metabolite 3-OH-BDE-47 (IC_50_ = 0.24 μM) showed a similar binding capacity with the positive control rosiglitazone, followed by 5-OH-BDE-47 with a calculated IC_50_ of 3.09 μM. In contrast 6-OH-BDE-47 and 6-OH-BDE-99 were not active ligands for PPARγ. The calculated IC_50_ for 5-OH-BDE 99 was 30 μM.

*Organophosphate/phosphite analogues of organotin*. As shown in Table 1, TBuP, TBEP, TPPi, and TPP were found to bind to the PPARγ LBD; however, the IC_50_ varied greatly between the compounds. TBuP (IC_50_ = 137 μM) and TBEP (IC_50_ = 103 μM) were two orders of magnitude less potent than TBT (IC_50_ = 0.3 μM). However, we also observed that TBuP could completely inhibit the binding between the probe and the PPARγ LBD at the high concentration (2,500 μM; see Supplemental Material, Figure S7). TPPi was much less potent at binding than TPP (IC_50_ = 40 μM) and TPT (IC_50_ = 1.72 μM) with an IC_50_ > 1,250 μM.

**Figure 2:**
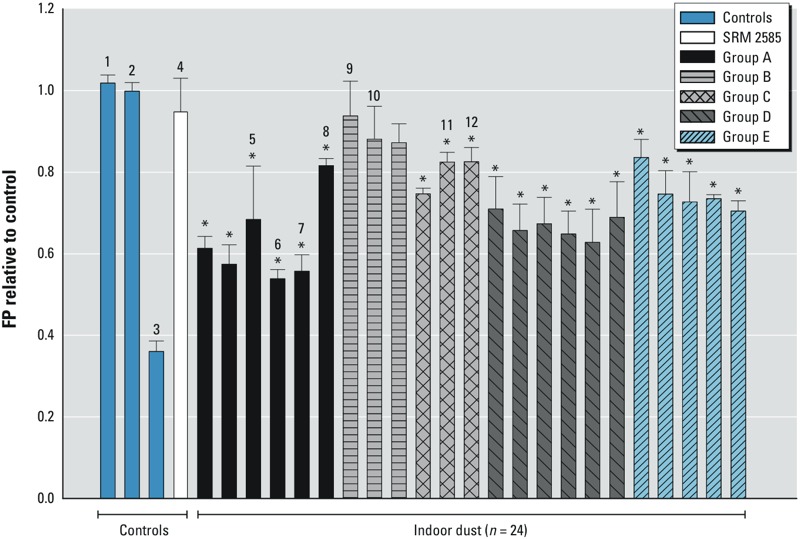
Fluorescence polarization (FP) value (mP) of 24 dust samples with a concentration of 3 mg dust/mL relative to the procedure dust blank in 40 μL of 38 nM PPARγ LBD and 1.25 nM PPARγ Green. Treatment: 1, DMSO control; 2, procedure blank; 3, positive control (12.5 μM rosiglitazone); 4, SRM 2585; 6, dust sample 6 used for dose–response; 5 and 7–12; dust extracts used in bioactivation. Values shown are the mean ± SD of the triplicates. *p < 0.05.

*Binding activity of dust samples*. Significant PPARγ binding activity of the dust samples at a concentration of 3 mg DEQ/mL was observed for 21 of the 24 dust samples tested (Figure 2). No significant binding was observed for SRM 2585. High variability was observed between the dust samples. Ten of the dust extracts competitively inhibited the binding between the PPARγ LBD and PPARγ Green by more than 40% of the control. The binding potency of those dust extracts was only slightly lower than the positive control (12.5 μM of rosiglitazone), which could completely inhibit the binding between the PPARγ LBD and PPARγ Green probe. Dust sample 6, which demonstrated a high binding potency, was selected to quantitatively evaluate the binding potency, and a clear dose–response relationship was observed (see Supplemental Material, Figure S8A). The calculated IC_50_ of dust sample 6 was approximately 0.37 mg DEQ/mL. We also observed differences in binding potency among dust extracts from different sources. For example, the dust extracts from Groups A and D, which were collected from main living areas in homes, showed a higher binding affinity with PPARγ than other groups (Figure 2). In contrast, the Group B samples collected from gymnastic studios did not show any obvious binding.

**Figure 3:**
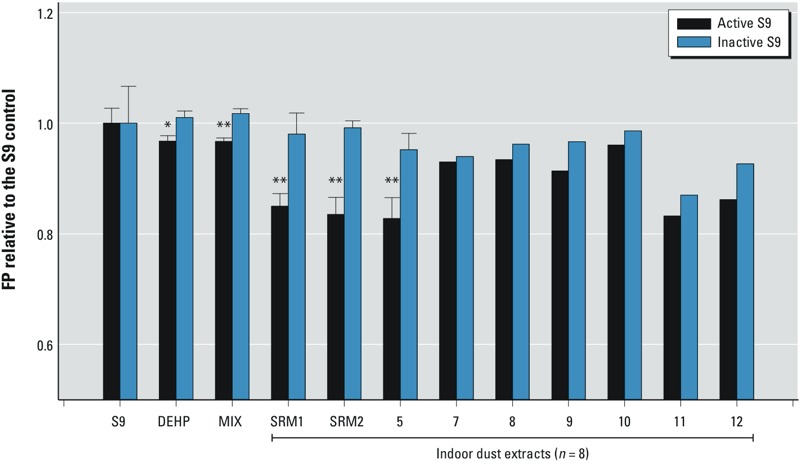
Competitive PPARγ binding potency of rat liver S9 control, DEHP, MIX SRM 2585, and seven other dust samples (100 mg) by incubation with either S9 or inactive S9 fraction (1 mg protein/mL) in a final volume of 3 mL. All data were normalized to the mP of S9 control. MIX includes 5 μM each of FM550, ITP, BDE‑47, BDE‑99, and DEHP. SRM1 and SRM2 represent the incubation of SRM 2585 with rat liver S9 and human liver S9, respectively. The dosing concentrations were 100 μM for DEHP, 2 μM for MIX, 3 mg DEQ/mL for SRM1 and SRM2, and 6 mg DEQ/mL for other dust samples. Values shown are the mean ± SD of the triplicates; samples without error bars represent only one incubated sample. *p < 0.05. **p < 0.01.

*Bioactivated dust samples*. We observed no difference in ligand activity between the extracts of active and inactive S9 fractions alone (i.e., S9 control; Figure 3). The potency of PPARγ binding was slightly increased after bioactivation of 100 μM DEHP (*n* = 3), and the bioactivated MIX (*n* = 3) showed an approximate 5% increase in binding (i.e., ~ 10 mP). Bioactivated SRM 2585 using rat liver S9 fraction (SRM1) was significantly more potent with an approximately 16% (i.e., 40 mP) increase in inhibition. A similar increase (~ 18%) was observed for SRM 2585 incubated with the human liver S9 fraction (SRM2), suggesting similar bioactivation effects on PPARγ binding. In dust sample 5, a significant increase (~ 13%) in binding was also found after bioactivation. A slight increase (3–10%) was also observed in other incubated dust samples. A paired *t*-test including all the dust samples with single incubations revealed that bioactivated dust samples showed significantly stronger binding potency with PPARγ than dust samples incubated with the inactive S9 fraction (*p* < 0.01). To quantitatively observe the change with different doses, we conducted a dose–response analysis to investigate the binding potency of the MIX, bioactivated MIX, and SRM 2585. We observed a partial dose–response curve because the dust matrix or S9 co-extracts interfered with polarization at high doses (see Supplemental Material, Figure S2C,D). As shown in Supplemental Material, Figure S8B, higher inhibition potency was observed for the bioactivated MIX in the dynamic range of the dose–response curve. Bioactivated SRM 2585 also showed a dose–response curve (see Supplemental Material, Figure S8C), although no inhibition was observed for the nonactivated extract (Figure 2A). Thus, our data indicate that PPARγ binding potency of dust samples increases after metabolism.

## Discussion

PPARγ is a master nuclear receptor that regulates lipid metabolism, cell proliferation signal transduction, apoptosis, and differentiation. Until now, few environmental contaminants have been shown to significantly bind and activate PPARγ signaling. This study was designed to test the PPARγ binding potency of several major FRs, including FM550 and PBDEs along with their metabolites using a ligand-binding competitor assay. Further, we examined the PPARγ binding of semivolatile organic compounds that are structurally similar to known PPARγ agonists, such as organotins and halogenated bisphenols. The binding potency of house dust samples and their bioactivated extracts was also examined. To our knowledge, very few studies have been conducted to investigate PPARγ activity in environmentally relevant dust samples. However, it should be noted that no definitive conclusions can be drawn from this PPARγ binding data as to whether these samples would lead to transactivation of PPARγ.

The data presented here are consistent with data reported in previous studies based on a luciferase gene reporter cell line assay. For example, the *in vitro* binding of FM550 and its components were consistent with the Cos-7 luciferase reporter assay, which indicated that TPP was the major contributor to the PPARγ binding in the commercial mixtures (Pillai et al. 2014). The relative potency of TBBPA and TCBPA tested in the present study was also similar to the results of the HGELN-GAL-PPAR assay reported by Riu et al. (2011). Therefore, our study indicates that this direct protein–ligand binding competitor assay can be used as an effective alternative method in the early screening of PPARγ ligands.

We found that several of the tested chemicals and their metabolites could competitively bind with the PPARγ LBD, but the calculated IC_50_ values and *K*_d_ of the tested compounds with the PPARγ LBD varied considerably. Most of the previously reported potential PPARγ ligands (e.g., TBBPA, TCBPA, TBMEHP), TBT, and TPT) were confirmed in this study using a different bioassay. To the best of our knowledge, many of the compounds tested here, including halogenated phenols, several hydroxylated metabolites of PBDEs and FM550, TBuP, TBEP, and TPPi were shown for the first time to have PPARγ binding activity. Although some of the tested compounds (e.g., TBEP and TBuP) showed weaker PPARγ binding potency, these compounds may yet be of great concern because of their ubiquitous detection in indoor environments, with levels up to micrograms to milligrams per gram of dust (Van den Eede et al. 2011).

Our study also revealed that metabolites of many FRs can be more potent than their parent compounds. There have been increased public health concerns about PBDEs for decades because of their potential disruption of thyroid hormone regulation and neurodevelopment (Noyes et al. 2011). BDE-47 and BDE-99, predominant components of the banned pentaBDE commercial mixture that are still widely detected in the environment, did not show strong binding potency to PPARγ. However, OH-BDEs, which are formed through cytochrome P450-mediated oxidative metabolism of BDEs, were found to be potent PPARγ ligands in the present study. The metabolite 3-OH-BDE-47 exhibited a comparable binding potency to the drug rosigilitazone. 5-HO-BDE-47, which is one of the most abundant metabolites of BDE-47 (Qiu et al. 2007), also showed a very strong binding potency. Due to the high potency of OH-BDEs in PPARγ signaling disruption, their role should be investigated. Although the other two major components of FM550—TBB and TBPH—did not show any binding activity, their metabolites (TBMEHP and TBBA) can be potent ligands of PPARγ. Although TBMEHP was not readily metabolized from its parent TBPH by enzymes in human hepatic S9 fractions or microsomes in our previous *in vitro* study (Roberts et al. 2012), the other major metabolites (i.e., DPP and TBBA) have been frequently identified in human urine samples (Cooper et al. 2011; Hoffman et al. 2014; Meeker et al. 2013). To date, little toxicological information has been reported for TBBA, and further studies should examine its potential to disrupt PPARγ.

Our results highlight several characteristics that may increase binding potency to PPARγ. First, halogenation, especially bromination, increases the potency of PPARγ binding, which was confirmed by the specific binding activities of halogenated phenols and bisphenols. The flame retardant 2,4,6-TBP showed a similar binding potency with TPP. Our structure–activity relationship experiments showed that the inhibition potency generally increased with increasing halogen molecular weight (i.e., iodine > bromine > chlorine > fluorine), which suggests that nonspecific hydrophobic interactions (i.e., Van der Waals force) with the PPARγ binding pocket favor binding. These findings are consistent with studies investigating thyroxine–transthyretin binding affinity and deiodination activity inhibition (Meerts et al. 2000). In the present study, we also observed a similar trend for TCBPA and TBBPA, which was consistent with a previous study suggesting that bulkier compounds bind more strongly with PPARγ (Riu et al. 2011). The IC_50_ of TB-MEHP was one order of magnitude lower than the IC_50_ of MEHP, which suggests that halogenation supports binding. All of these findings indicate that the large ligand binding pocket of PPARγ can readily accommodate the addition of bulky bromine or chlorine. Therefore, disruption of PPARγ signaling may be a major concern for FRs because a large number of FRs are halogenated. Second, we also found that the number of halogens and the position of the hydroxyl group affect PPARγ binding. In this study, we observed a dose–response relationship for BDE-47 but no binding for BDE-99. Suzuki et al. (2013) also observed a dose–response relationship between PPARγ2 and BDE-47 using a 5% induction concentration of 10 μM in a human osteosarcoma (U2OS) cell-based reporter assay, but they observed no activity for other BDEs. The variable IC_50_ values of BDEs and OH-BDEs we found in the present study suggest that the OH-BDEs with a *meta* hydroxyl group exhibited stronger PPARγ binding potency than OH-BDEs with an *ortho*-substituted hydroxyl group. Among the OH-BDEs tested, 3-OH-BDE-47 had the most similar structure to that of the known PPARγ agonist TBBPA, with a *meta*-substituted hydroxyl group and two adjacent bromine atoms. Finally, we observed that the PPARγ binding potency differed greatly for chemicals with similar structures. Organophosphates were more potent than organophosphites, but both were much less potent than organotins, which suggests that some other chemical feature, perhaps the electron density of the tin atom, might play an important role in the binding. Alternatively, this also may be related to the relative solubilities of the compounds.

To date, few toxicological studies have investigated potential health effects from environmentally relevant house dust samples; studies using dust samples are more insightful for human exposure than are exposures to pure chemicals. Because many semivolatile organic compounds bind to dust in the indoor environment, in this study we tested dust samples for PPARγ binding potency. Binding activity was observed in most of the dust samples (21 of 24 dust samples), and differences were observed between groups of dust extracts. To date, the chemical composition of the dust samples from different sources has not been characterized. In a previous study (Carignan et al. 2013a), concentrations of FRs—particularly PBDEs in the dust from a gymnasium—were at least one order of magnitude higher than levels in residential dust, suggesting that those FRs might not be primary contributors to the PPARγ binding. However, the small sample size and heterogeneity of the house dust samples in the present study prevent any solid conclusions from being made. In addition, the binding potency of the house dust in our study might be underestimated due to FP interference from the dust matrix. Because young children spend most of their time indoors and are exposed to house dust via frequent hand-to-mouth behavior, tests on dust samples are needed to determine the public health concerns for exposures to contaminant mixtures present in dust. The U.S. EPA (2009) estimated that children ingest between 50 and 100 mg/dust per day. In the present study, we found an IC_50_ of 0.37 mg DEQ/mL for one of the most potent dust samples. Therefore, our data suggest that environmentally relevant dust exposures might interact with PPARγ *in vivo*.

We investigated the bioactivation of dust samples to increase understanding of the potential activity *in vivo* following metabolism. We observed stronger binding potency in the bioactivated dust samples compared with the raw dust extracts. Bioactivation could transform the hydrophobic chemicals into more polar metabolites by adding, for example, a hydroxide or carboxylate, which might increase the binding interaction with the LBD through hydrogen bonds. It might be possible that compounds in dust, such as TBB, TBPH, PBDEs, and DEHP, could be metabolized to PPARγ-active ligands after incubation, which was supported by the increased binding potency of the prepared MIX containing these chemicals. Although the effect of bioactivation was less than approximately 20%, it is possible that *in vivo* metabolism would lead to higher binding activity. Chemicals in the human body would have a half-life that is longer than our 2-hr incubation, which would lead to longer contact time with xenobiotic-metabolizing systems in the body. Therefore, bioactivation should be considered when evaluating potency of environmental chemicals and potential human health risks.

## Conclusion

Results of the present study indicate that many of the tested compounds or metabolites are potential PPARγ ligands. Significant binding activity of environmentally relevant dust samples was observed with high frequency. We also observed that bioactivation could increase the binding potency of chemical mixtures in the ingested dust. Further work is needed to determine which components in the dust samples are acting as ligands. A limitation of this study is that ligand binding does not necessarily indicate agonism of the receptor, leading to transcriptional events. Ligands can be agonists (full or partial) or competitive antagonists. To confirm the health effects of the identified PPARγ ligands, further studies using cell-based reporter assays that can distinguish between agonism and antagonism should be conducted.

## Supplemental Material

(2.6 MB) PDF
