# Supplementary material for: Characterizing the Peroxisome Proliferator-Activated Receptor (PPARγ) Ligand Binding Potential of Several Major Flame Retardants, Their Metabolites, and Chemical Mixtures in House Dust
Source: Environ Health Perspect. 2014 Oct 14;123(2):166–72. doi: 10.1289/ehp.1408522 (PMC4314249; doi:10.1289/ehp.1408522)
Supplement: (2.6 MB) PDF [file ehp.1408522.s001.508.pdf]

Environmental Health Perspectives doi:10.1289/ehp.1408522

Supplemental Material

Characterizing the Peroxisome Proliferator–Activated Receptor (PPARγ) Ligand Binding Potential of Several Major Flame Retardants, Their Metabolites, and Chemical Mixtures in House Dust

M. Fang, T.F. Webster, P.L. Ferguson, and H.M. Stapleton

Abbreviations

2,4,6-TFP – 2,4,6-trifluorophenol;
2,4,6-TCP – 2,4,6,-trichlorophenol;
2,4,6-TBP – 2,4,6-tribromophenol;
2,4,6-TIP – 2,4,6-triiodophenol;
BDE47 – 2,2',4,4'-tetrabromodiphenyl ether;
BDE99 – 2,2′,4,4′,5-pentabromodiphenyl ether;
DEHP – di-(2-ethylhexyl) phthalate;
DEQ – dust equivalent quantity;
DPP – biphenyl phosphate;
DTT – DL-dithiothreitol;
FB – fluorescence background;
FM550 – Firemaster 550;
FP – fluorescence polarization;
ITP – isopropylated triphenyl phosphate;
LBD – ligand-binding domain;
MEHP – mono(2-ethylhexyl) phthalate;
MP – polarization value;
PAHs – polycyclic aromatic hydrocarbon;
PPARγ – peroxisome proliferator activated receptor γ;
PBDE – polybrominated diphenyl ether;
PentaBDE – pentabrominated diphenyl ether;
SVOCs – semi-volatile organic compounds;
TBB – 2-ethylhexyl-2,3,4,5-tetrabromobenzoate;
TBBA – 2,3,4,5-tetrabromo benzoic acid;
TBBPA – 2,2′,6,6′-Tetrabromobisphenol A;
TBEP – tris(2-butoxyethyl) phosphate;
TBMEHP – tetrabromo mono(2-ethylhexyl)phthalate;
TBPH – bis(2-ethylhexyl) tetrabromophthalate;
TBT – tributyltin chloride;
TBuP – tributylphosphate;
TCBPA – 3,3’,5,5’-tetrachlorobisphenol A;
TPP – triphenyl phosphate;
TPT – triphenyltin chloride;
TPPi – triphenylphosphite;

PPARγ Competitive Binding Assay and Quality Assurance/Quality Control

PPARγ competitive binding assay

Fluorescence polarization (FP) assays allow ligand binding to be quantified in a homogeneous format without perturbing equilibrium by physical separation of bound vs. free ligand, which is advantageous for the measurement of low-affinity interactions (Rossi and Taylor 2011). In this study, a commercially available high-throughput ligand binding assay (PolarScreen™ PPARγ-competitor assay kit, Invitrogen) was used to investigate the binding potency of tested compounds to PPARγ LBD. The kit uses the human-derived recombinant PPARγ-LBD tagged with a N-terminal GST-tag and a selective fluorescent PPARγ ligand (PPARγ Green) in a 384-well plate (Corning Thermowell GOLD 3756). The test compounds were dissolved in DMSO and dosed into each well with a final volume of 40 μL containing 38 nM PPARγ LBD and 1.25 nM PPAR-Green as recommended by the protocol. We used 3% DMSO in the final incubation mixture to reduce potential effects on polarization by the solvent. The well plate was mixed and incubated for 3 hours at room temperature to reach equilibrium. A SpectraMax M5 plate reader was used in polarization mode with 485 nm excitation and 535 nm emission wavelenth. To measure ligand binding, we quantified polarization (mP) of the bound protein using the following equation:

$$mP = 10^3*(I_p-I_s)/(I_p+I_s) \qquad [1]$$

where I_p and I_s are the fluorescence intensity of emissions that are parallel (P) and perpendicular (S) to the excitation light; respectively (Rossi and Taylor 2011).

Quality assurance/quality control

For each dose level of tested compounds, triplicate samples were prepared and each well was read five times in the plate reader. DMSO and rosiglitazone were run alongside each batch as a control and positive control, respectively. For the dust samples, a procedural blank was prepared and preceded alongside the dust extracts to examine the background contamination. Since variability of FP was observed across the batches, normalization to DMSO control was conducted when comparing between batches. The potent ligands such as 3-OH-BDEs were tested three times at different days with different well plates.

Operation of Gel Permeation Chromatography

The flow rate was set to 10 mL/min and DCM was used as the mobile phase. To test the elution profile, several compounds ranging from small MW halogenated phenols to large MW TBPH were loaded onto GPC and fractions were collected at 0–10 min, and then 2 min for each fraction until 30 mins. As shown in Supplemental Material, Table S1, most of the chemicals were eluted after 14 mins except TBPH. To recover the highest amount from the mixture, a fraction from 12–28 min was collected, concentrated, and solvent exchanged into DMSO.

Bioactivation of Dust Samples

The incubation method was modified based on previous studies (Montaño et al. 2012) and the flow chart was shown in Figure S3. To increase the amount of the metabolites, incubation was up-scaled and performed in a borosilicate glass tube in 3 mL phosphate buffer (PB, 100 mM, pH 7.4), S9 fraction (1 mg protein/mL), and dust extract in ~30 µL DMSO (~33 mg dust/mL PB). 10 mM DTT and 6 mM magnesium chloride were added. After 5 minutes pre-incubation in a shaking water bath at 37℃, the reaction was initiated with the addition of 50 µL of 60 mM

NADPH in PB. Additional 50 μL of 60 mM NADPH in PB were added after 60 mins. Metabolism was stopped after 120 minutes by denaturation of microsomal protein with 150 μL of ice-cold 6M HCl. An additional sample for each dust extract was incubated with inactive S9 by 150 μL of ice-cold 6M HCl before incubation and run alongside as the comparison control.

The challenges in the bioactivation of dust samples for the PPARγ binding assay includes the low metabolic rate, interference from coextracts in S9 fraction, and FB of dust (Montaño et al. 2013). In our preliminary study, we found that S9 extract could interfere with the PPARγ polarization assay [see Supplemental Material, Figure 2 (b), 2 (c), 2 (d), and Figure S9], which might be caused by the lipids which can work as natural ligands for PPARγ. Therefore, further cleanup was needed to remove the coextracts that interfere with PPARγ binding. In this study, the extraction and cleanup method for the metabolites was modified according to a recently published low-fat method for metabolite extraction, including dextran assisted extraction and lipid removal agent (LRA) cleanup (Montaño et al. 2012). Briefly, the fat was flocculated with 50 μL of 10% dextran with 150 μL of 6 M HCl previously added to facilitate the flocculation. Metabolites were extracted with 2 × 2 mL ethyl acetate and 1× 2 mL hexane : methyl tert-butyl ether (1:1, v/v). TBBA, TBBPA and MEHP were tested for the recovery of dextran and LRA cleanup. Good recovery (> 80%) was observed in the dextran assisted extraction and no elution of any compound in the LRA. Therefore, only dextran extraction was used in the study.

The low metabolic rate does not generate sufficient metabolites to activate the PPARγ binding and further concentration of the metabolites was needed. However, the dust matrix will be concentrated and cause fairly high fluorescence background to interfere with the PPARγ binding. Thus, further cleanup of the dust matrix was still needed. In our previous studies, we found that the phenolic extraction could retain most of the dust matrix in the organic solvent. Furthermore,

most of the known PPARγ agonists or antagonists are the chemicals with polar groups such as –OH, –COOH, and $–NH_2$. If we hypothesize that charged chemicals including phenolic or carboxylic metabolites were the major metabolites, it would be possible to reduce the dust matrix without losing most of the metabolites as a tradeoff. In this study, the extracts from dextran assisted liquid-liquid extraction was blown down to near dryness under nitrogen gas and the sample was reconstituted in 1 mL DCM, which was then extracted with 3×1 mL deionized water with a pH of ~13. The pool of the 3 mL water was then acidified with 6 M HCl to pH < 3 and extracted with 2 × 2 mL ethyl acetate and 1 × 2 mL hexane : methyl tert-butyl ether (1:1, v/v). The final extracts were dried and reconstituted with 200 µL DMSO. As shown in Figure S2 (b), the phenolic extraction can reduce the matrix background of the dust extracts to the level which was only slightly above the DMSO control and would not greatly interfere with the binding assay. The recoveries of tested compounds such as TBBPA, MEHP and TBBA were > 85% using the above-mentioned method. Therefore, the combination of dextran-assisted extraction and phenolic extraction make the PPARγ binding assay feasible for the bioactivated dust extracts.

Performance of the Bioactivation of Dust

The challenges in the bioactivation of dust samples for the PPARγ binding assay includes the low metabolic rate, interference from coextracts in S9 fraction, and FB of dust. As shown in Supplemental Material Figure S9, the natural ligands such as fatty acid in the S9 fraction can competitively inhibit ~20% of the binding between PPARγ-LBD and PPARγ Green at a concentration of 250 µg protein/mL. Dextran was used to selectively remove the lipid during extraction (Montaño et al. 2012). The result showed the dextran assisted extraction could partially remove the interference from the coextracts, which was close to 90% of the DMSO control. The application of incubation with inactive S9 running alongside as control could correct

this interference. Phenolic extraction could further reduce the FP background by approximately five times than that of the raw extract, leaving the FP of dust with a concentration of 6 mg DEQ/mL close to DMSO control (see Supplemental Material Figure S2 (b)). As shown in Supplemental Material Figure S3, MEHP could be formed by the incubation of dust with S9 fraction and the formation rate was approximately 70 pmol/mg protein/min, which was slightly higher than that of 25 μM DEHP pure chemical. However, the formation rate was decreased when the dosing amount of dust was 100 mg/mL, which might due to the decrease of enzyme activity caused by the impurities in dust extracts. Therefore, we used ~33 mg DEQ/mL throughout the incubation experiment in this study to maintain a high metabolic rate.

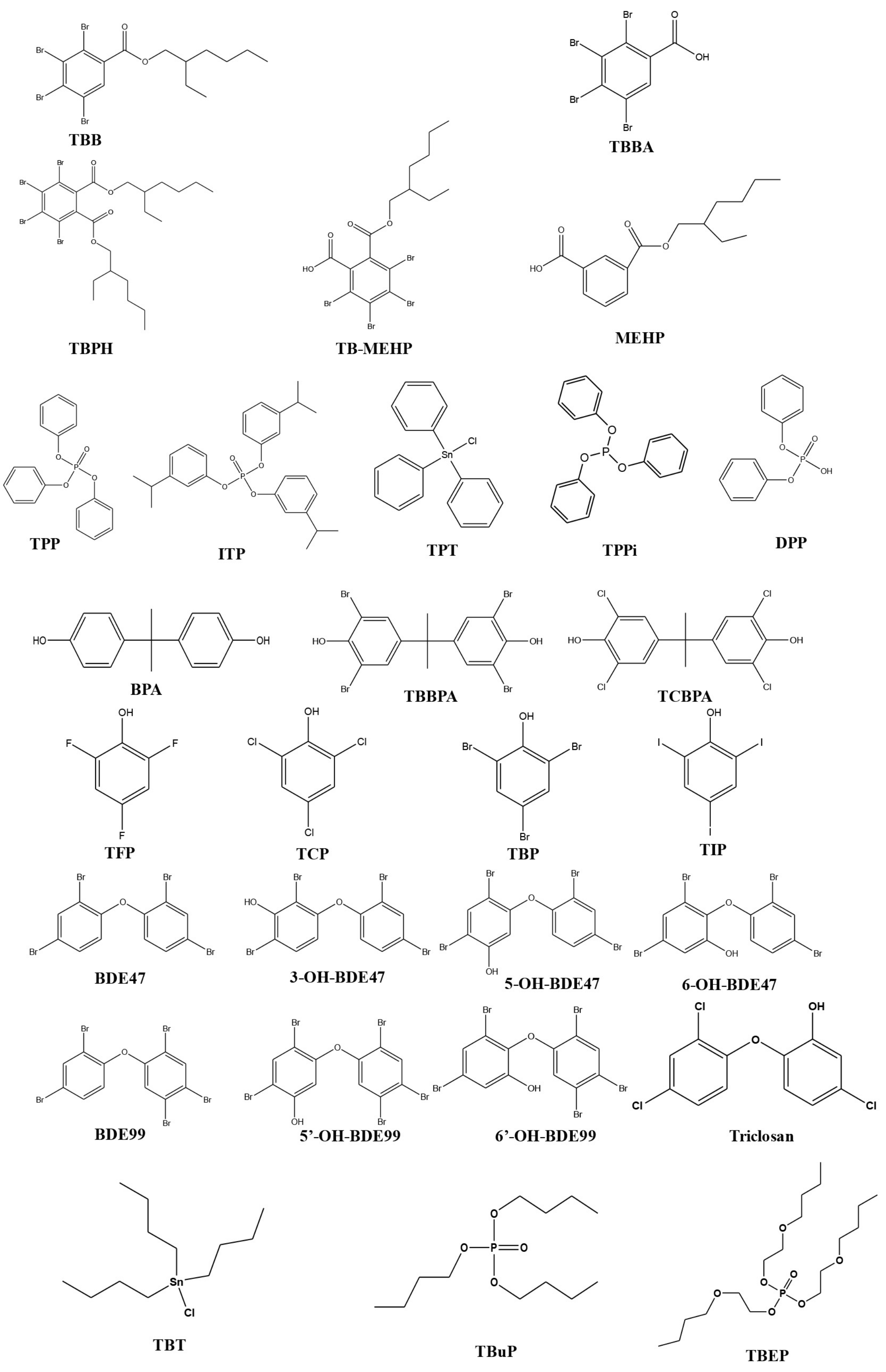

Figure S1. Structures of the flame retardants and their major metabolites tested in this study.

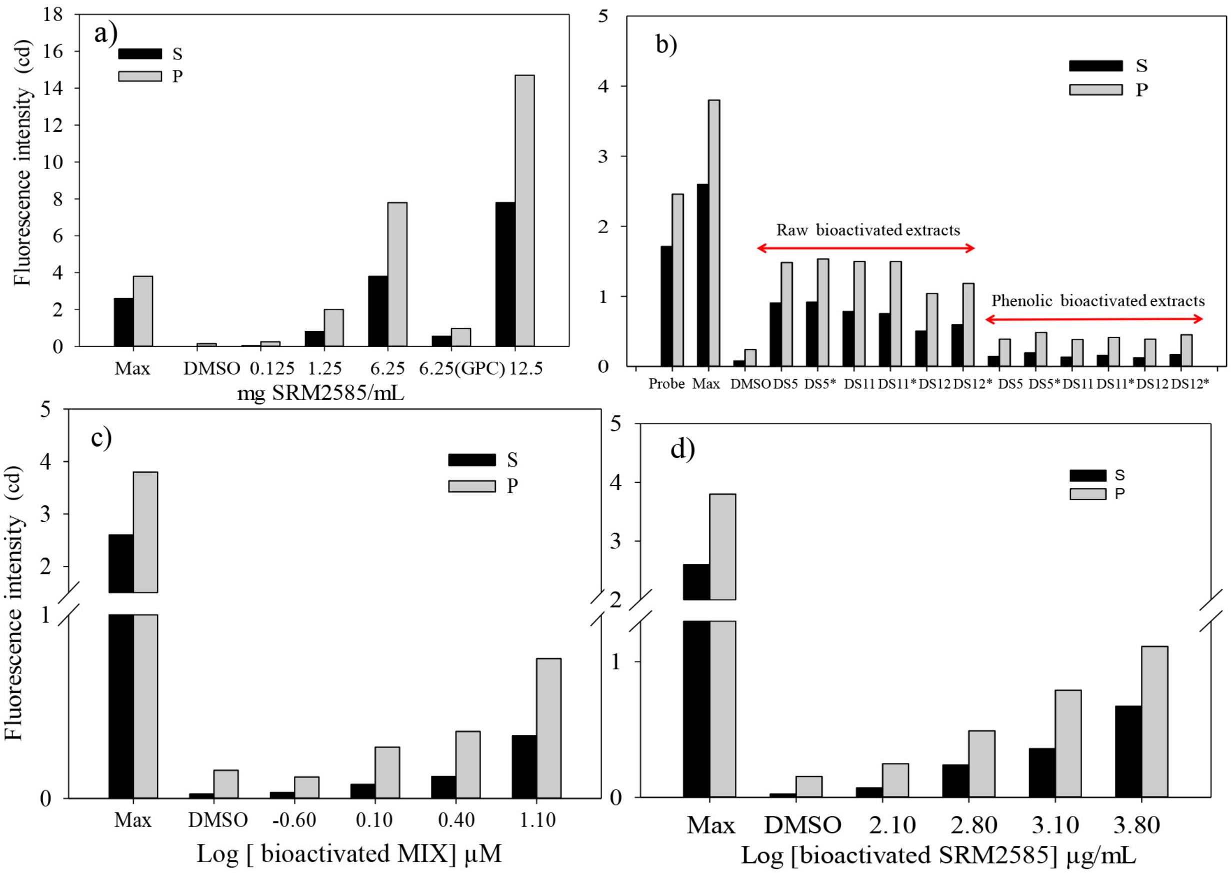

Figure S2. The fluorescence interference from dust matrix and rat liver S9 fraction coextracts before and after cleanup treatments. In details, the figures show fluorescence intensity (cd) of emission parallel (P) to the excitation plane, and fluorescence intensity perpendicular (S) to the excitation plane of: (a) SRM 2585 prior to and post GPC treatment; (b) raw bioactivated dust extracts (6 mg DEQ/mL) and phenolic fraction; (c) bioactivated MIX; and (d) bioactivated SRM2585 with different concentrations. The assay was conducted by dosing dust extract to the buffer solution without PPARγ-LBD and PPAR-Green. DMSO represents the control without PPARγ-LBD and PPAR-Green. Max represents the DMSO with PPARγ-LBD and PPAR-Green. Probe represents PPAR-Green only. The labels with "*" in b) represent dust incubated with active S9 fraction. Those values represent average of the triplicates and error bar represents standard deviation.

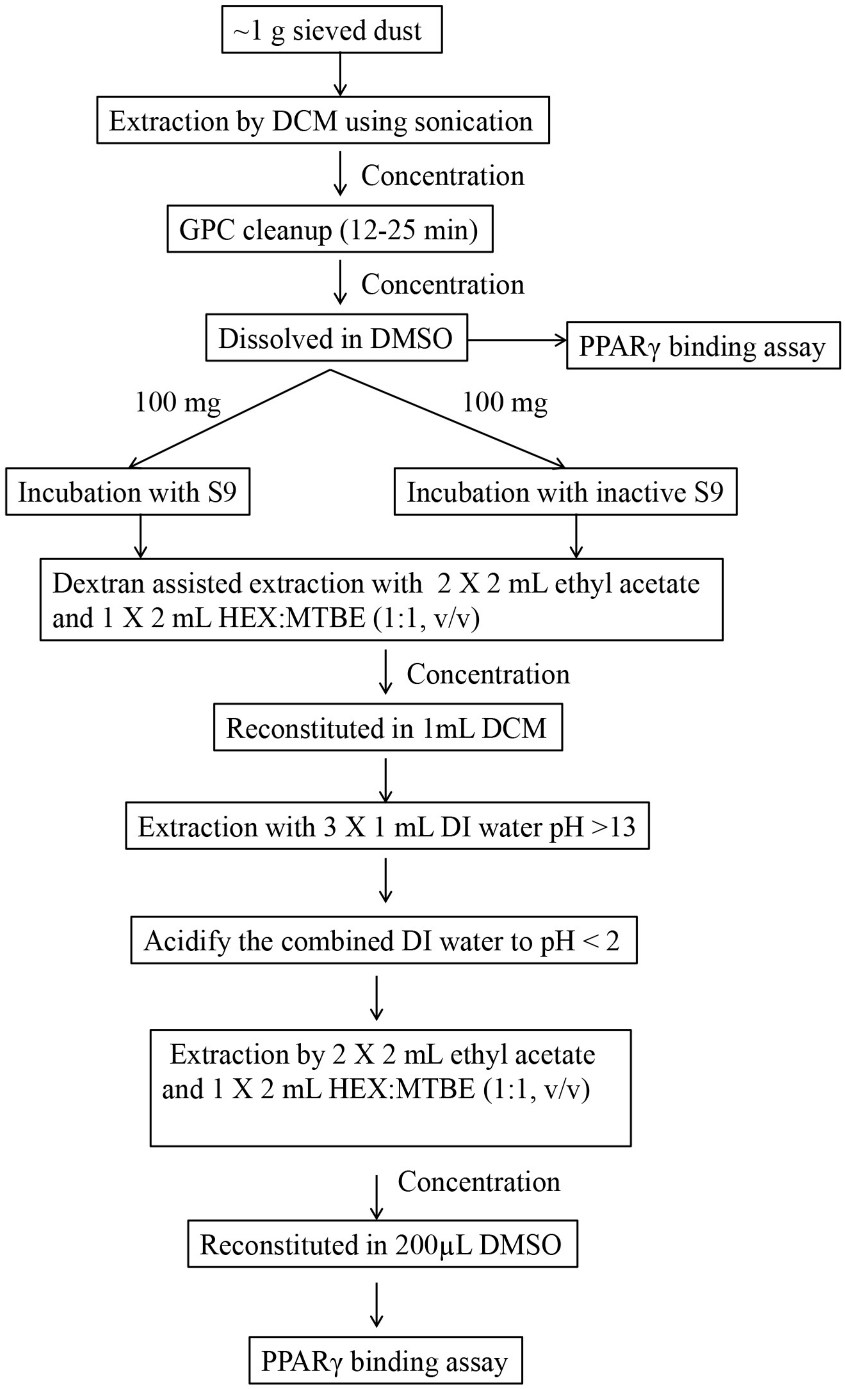

Figure S3. Flow chart of incubation, extraction, and cleanup steps for dust bioactivation experiments using rat liver S9 fractions.

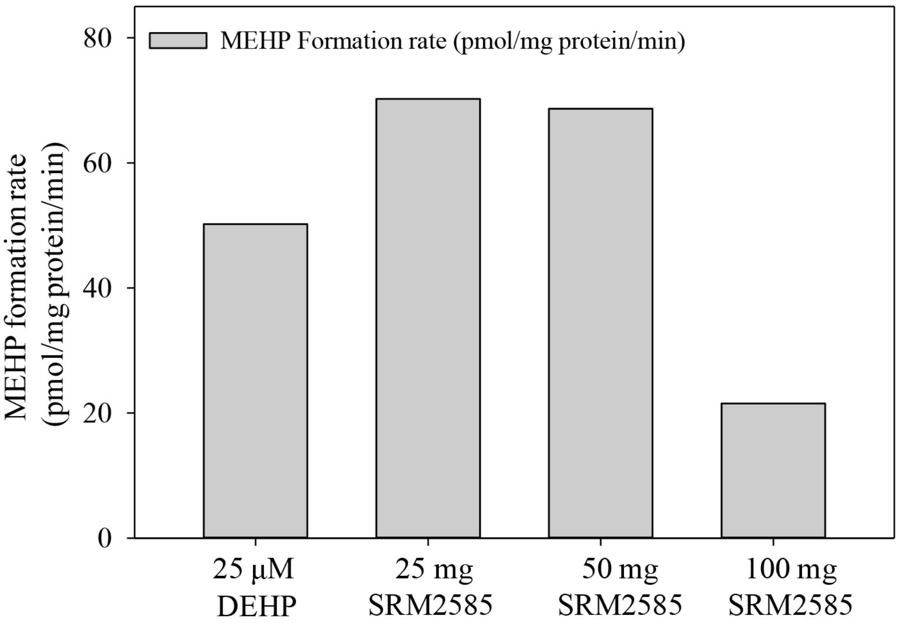

Figure S4. MEHP formation rate in incubations with either pure DEHP (25µM) or increasing concentrations of an extract of indoor dust SRM 2585. Values represent average of the triplicates and error bar represents standard deviation.

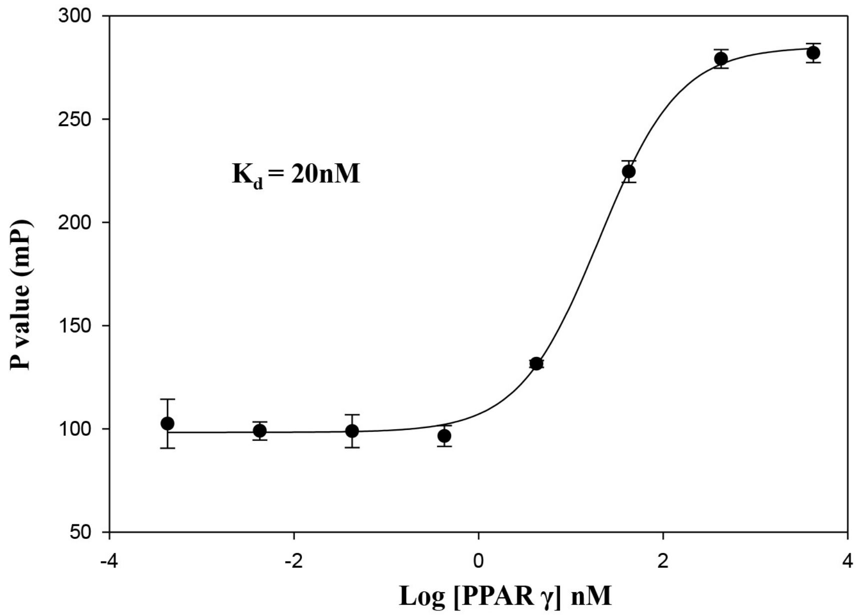

Figure S5. Fluorescence polarization value (mP) of 1.25 nM PPAR-Green as a function of added PPAR γ LBD concentration. Values represent average of the triplicates and error bar represents standard deviation.

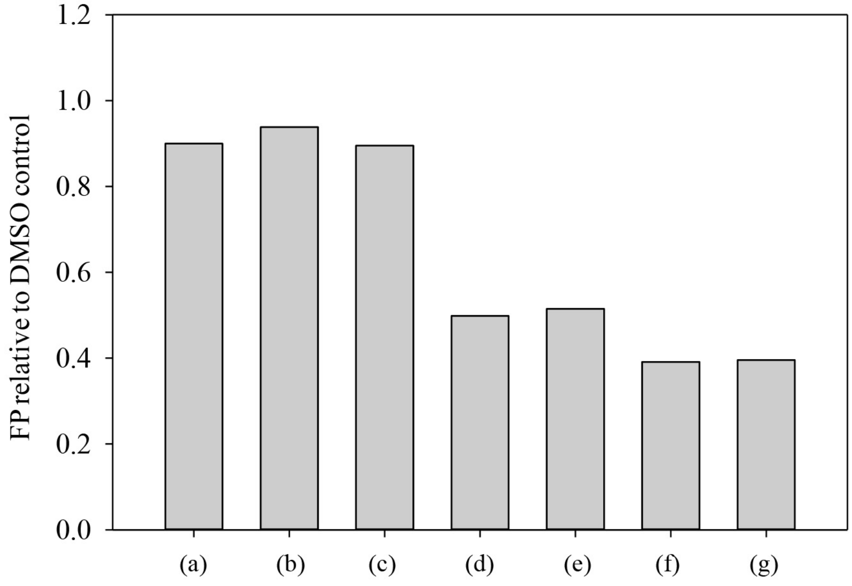

Figure S6. Competitive PPARγ binding potency of (a) 1.25 mg SRM2585 (cleaned by GPC)/mL; (b) 0.125 mg SRM2585/mL; (c) 12.5 mg SRM2585/mL spiked with 12.5 μM Rosi.; (d) 1.25 mg SRM2585/mL spiked with 12.5 μM Rosi.; (e) 0.125 mg SRM2585/mL spiked with 12.5 μM Rosi.; (f) 0.0125 mg SRM2585/mL spiked with 12.5 μM Rosi.; and (g) 12.5 μM Rosi. relative to the DMSO control. Values represent average of the triplicates and error bar represents standard deviation.

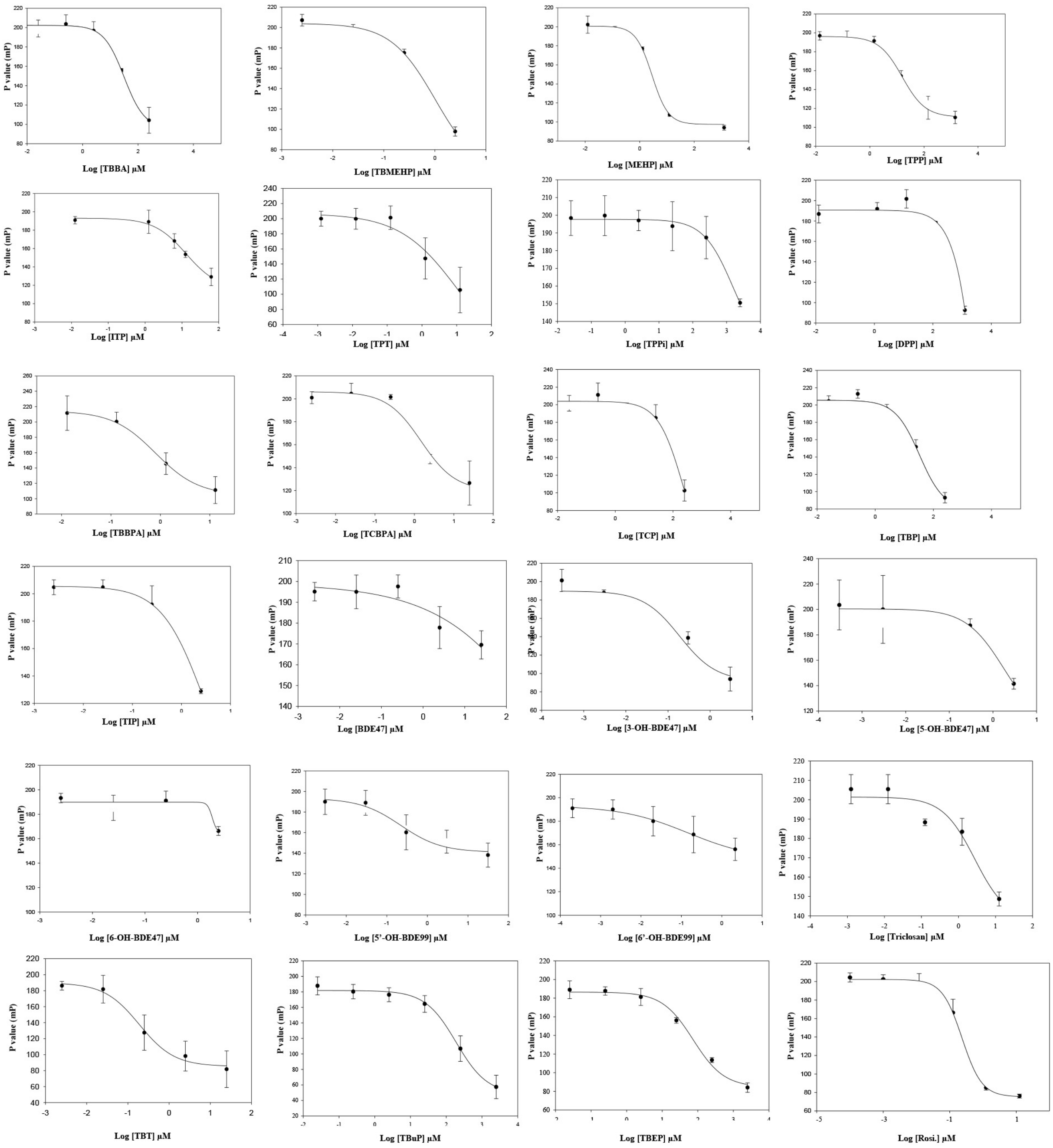

Figure S7. Dose response curve of tested flame retardants and their major metabolites. All assays were done with triplicates in 40µL of 38 nM PPARγ LBD and 1.25 nM PPAR-Green. Values represent average of the triplicates and error bar represents standard deviation.

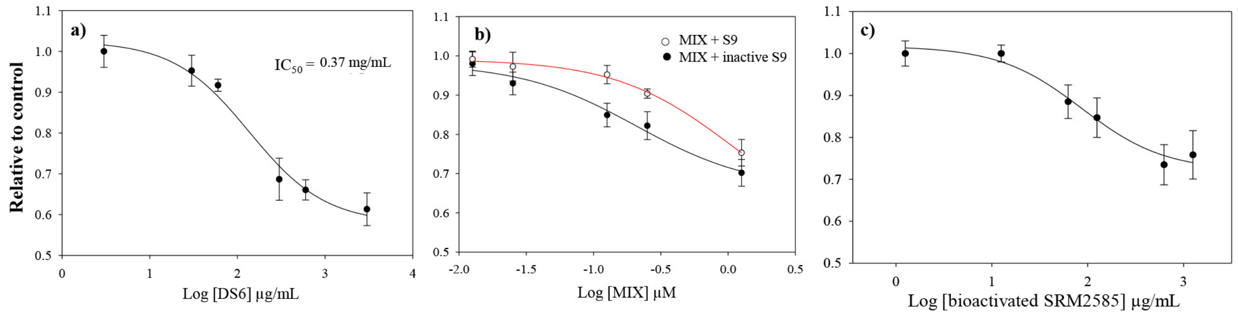

Figure S8. Dose response curve of PPAR• ligand binding using a) an indoor dust sample (DS6); b) MIX incubated with active and inactive S9 fraction; c) bioactivated SRM2585. MIX includes 5 µM FM550, ITP, BDE47, BDE99, and DEHP. Values represent average of the triplicates and error bar represents standard deviation.

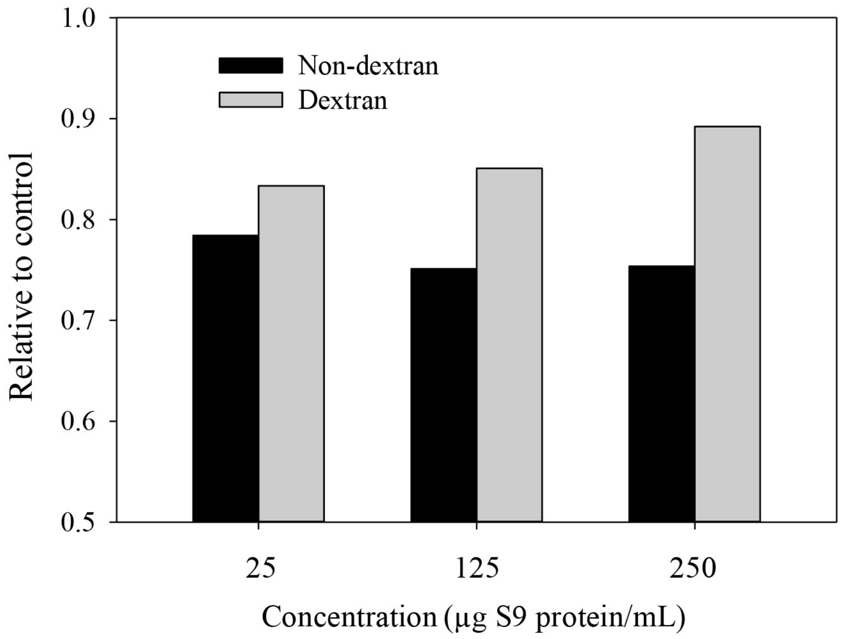

Figure S9. Competitive PPARγ binding potency of the raw coextracts and dextran assisted extracts from S9 fraction relative to the DMSO control. Values represent average of the triplicates and error bar represents standard deviation.

Table S1. Chemical analysis of related compounds in this study.

Analytes	I.S.	MRM/SIM	Fragmentor	Collision energy	Ionization
TBPH	F-BDE69	463			GC/ECNI-MS
13C BPA		239.1-223.1<br>239.1-75	120<br>120	5<br>35	ESI negative
BPA	13C BPA	227.1-212.1<br>227.1-133	120<br>120	15<br>25	ESI negative
TBBPA	13C TBBPA	543-447.8<br>543-445.8	160<br>160	45<br>35	ESI negative
13C TBBPA		555-459.8	160	35	ESI negative
13C TRICLOSAN		301-35.1	80	10	ESI negative
Triclosan	13C TRICLOSAN	289-35.1<br>287-35.1	80<br>80	10<br>10	ESI negative
2,4,6-TBP	13C TBBPA	330.8-79<br>328.8-79	120<br>120	40<br>40	ESI negative
2,4-Dichlorophenol	13C TBBPA	161-125.1<br>161-34.9	80<br>80	10<br>18	ESI negative
4-Monochlorophenol	13C TBBPA	127-34.9	80	15	ESI negative
DEHP	TIBA	391.4-149.1	80	24	ESI negative
MEHP	TIBA	277-134<br>277-127	120<br>120	8<br>8	ESI negative
TBBA	TIBA	436.6-392.6<br>436.6-79	75<br>75	5<br>25	ESI negative
TIBA		498.7-454.8<br>498.7-127	75<br>75	5<br>15	ESI negative

Table S2. Elution profile of several pure compounds in GPC.

Compounds/time (min)	0-12	12-14	14-16	16-18	18-20	20-22	22-24	24-26	26-28
TBPH		73%	20%	7%					
BPA				77%	13%	5%	5%		
TBBPA			63%	34%	3%				
Tricolsan				91%	9%				
2,4,6-TBP					62%	33%	5%		
2,4-Dichlorophenol					76%	24%			
4-Monochlorophenol				11%	52%	19%	11%		
Triphenyltin chloride				11%	12%	15%	16%	23%	24%
Tributyltin chloride				26%	27%	20%	11%	9%	6%
Matrix	+++*	+++	++	+					

*Represents the darkness of the color observed in the fractions.

References

Rossi AM, Taylor CW. 2011. Analysis of protein-ligand interactions by fluorescence polarization. Nat Protoc 6(3).

Lin Q, Ruuska SE, Shaw NS, Dong D, Noy N. 1999. Ligand selectivity of the peroxisome proliferator-activated receptor alpha. Biochemistry 38(1): 185-190.

Montaño M, Cocco E, Guignard C, Marsh G, Hoffmann L, Bergman A, et al. 2012. New approaches to assess the transthyretin binding capacity of bioactivated thyroid hormone disruptors. Toxicol Sci 130(1): 94-105.

Montaño M, Weiss J, Hoffmann L, Gutleb AC, Murk AJ. 2013. Metabolic activation of nonpolar sediment extracts results in enhanced thyroid hormone disrupting potency. Environ Sci Technol 47(15): 8878-8886.
